# Collaboration can preserve the integrity of gold standard carbon data from forest inventories

**DOI:** 10.1073/pnas.2409263121

**Published:** 2024-09-05

**Authors:** Sara A. Goeking, Christopher W. Woodall, Renate Bush, Linda S. Heath

**Affiliations:** ^a^United States Department of Agriculture Forest Service, Research and Development, Inventory, Monitoring, and Assessment Research, Logan, UT 84321; ^b^United States Department of Agriculture Forest Service, Research and Development, Inventory, Monitoring, and Assessment Research, Durham, NH 03824; ^c^United States Department of Agriculture Forest Service, Research and Development, Inventory, Monitoring, and Assessment Research, Washington, DC 20250

We applaud the recent commentary (1) proposing robust evaluation of the scientific evidence underlying Nature-based Climate Solutions (NbCS). As demand for credible forest data intensifies due to NbCS proposals, carbon markets and researchers increasingly look to national forest inventories (NFIs) as a credible data source for unbiased appraisals. Although Novick et al. (1) advocate sharing public land NFI plot locations in the United States, unconditional data sharing introduces the possibility that known plot locations will no longer serve as an unbiased sample of forest management, carbon dynamics, and NbCS effectiveness (2).

NbCS have already consumed billions of dollars via private carbon markets, which introduce motivations for particular forest carbon outcomes. The nearly 100-year-old Forest Inventory and Analysis (FIA) program in the USDA Forest Service faces conflicting demands for open data from external entities with financial incentives to meet carbon market expectations and legal requirements to protect plot location confidentiality and data integrity (3). Beyond carbon, FIA data are foundational to forest assessments such as the 2022 Executive Order to inventory mature and old-growth forests, US compliance with European Union’s deforestation regulations, and the United Nations Global Stocktake. Thus, compromised data integrity would adversely affect not only NbCS appraisal but other national-scale assessments.

**Fig. 1.**
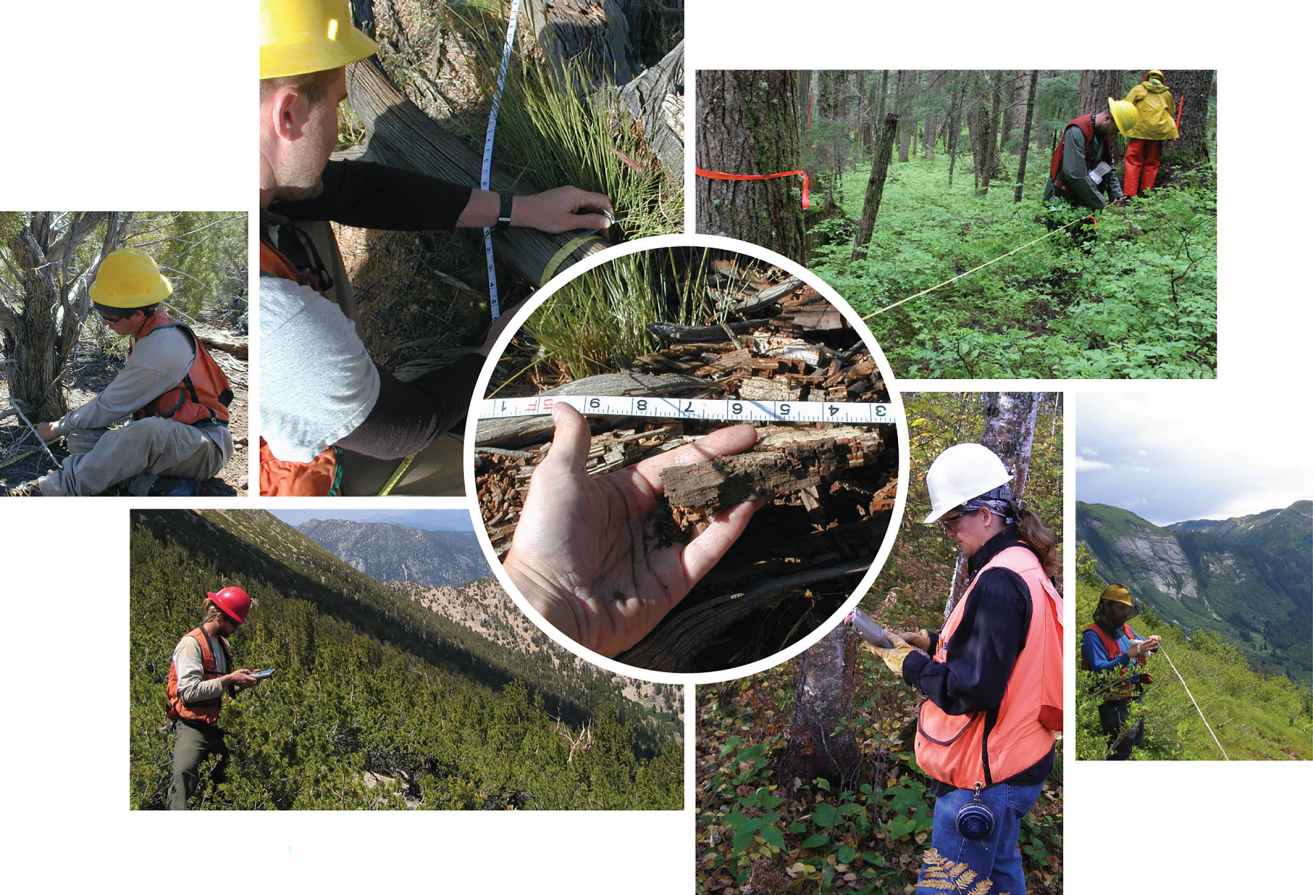
Forest Inventory and Analysis field crews collect forest measurements that allow quantification of various carbon pools, including those that cannot easily be assessed through remote sensing, throughout US forests on all land ownership categories and forest types.

As an alternative to disclosing plot locations, we support and encourage the establishment of off-grid FIA plots near eddy covariance flux towers (1). Such a “network of networks” (1) facilitates increased precision of scaled forest attributes from the FIA dataset as the foundation of NbCS quantification. This solution encourages collaboration, similar to partnerships between academic institutions and FIA that refined individual tree allometric equations in a flexible framework (4). FIA’s plot network and rich data stream can serve not only as calibration and validation mechanisms for a proliferation of third-party NbCS products, or even reconciliation of divergent land sector carbon budgets, but also as a national, statistically sound framework for NbCS science and applications (Fig. 1).

We encourage potential collaborators to leverage the network of networks proposal (1) in conjunction with the >99% of the FIA database that is already publicly available (https://www.fs.usda.gov/research/programs/fia#data-and-tools). Similar to other nations’ NFIs (2), FIA shares confidential data with collaborators who follow data security plans, do not publish data that would permit the identification of plot locations (3), and advance FIA’s mission as authorized by the US Congress (5). Annually, FIA responds to hundreds of data requests and supports thousands of data downloads (5), leading to insights into carbon dynamics (6–8), projections of future disturbance risk (9), and calibration or validation of widely used geospatial datasets (10).

We thank Novick et al. (1) for illuminating the need for increasing the scientific veracity of NbCS while empowering social response. We share these goals and foresee collaborations that creatively and transparently advance our collective ability to understand and apply NbCS in forests without compromising foundational datasets such as FIA and other nations’ forest inventories. As climate change progresses, the importance of maintaining FIA data credibility and integrity will grow alongside society’s need for these data (2).
